# Supplementary material for: Rapamycin Alternatively Modifies Mitochondrial Dynamics in Dendritic Cells to Reduce Kidney Ischemic Reperfusion Injury
Source: Int J Mol Sci. 2021 May 20;22(10):5386. doi: 10.3390/ijms22105386 (PMC8160749; doi:10.3390/ijms22105386)
Supplement: Supplementary file 1 [file ijms-22-05386-s001.zip › ijms-1209255-supplementary.pdf]

Rapamycin Alternatively Modifies Mitochondrial Dynamics in Dendritic Cells to Reduce Kidney Ischemic Reperfusion Injury

Maria Namwanje[1,2,5], Bijay Bisunke[2], Thomas V. Rousselle[5], Gene Lamanilao[5], Venkatadri S. Sunder[3], Elizabeth C. Patterson[5], Canan Kuscu[5], Cem Kuscu[5], Daniel Maluf[5], Manjari Kiran[3], Valeria Mas[5], James D. Eason[5] and Amandeep Bajwa[2,4,5,*]

[1]Department of Pediatrics, University of Tennessee Health Science Center, Memphis, TN, United States

[2]Department of Genetics, Genomics, and Informatics; College of Medicine, The University of Tennessee Health Science Center, Memphis, TN, USA.

[3]Department of Systems and Computational Biology, School of Life Sciences, University of Hyderabad, India

[4]Department of Microbiology, Immunology, and Biochemistry; College of Medicine, The University of Tennessee Health Science Center, Memphis, TN, USA.

[5]Transplant Research Institute, James D. Eason Transplant Institute, Department of Surgery, College of Medicine, The University of Tennessee Health Science Center, Memphis, TN, USA.

1. Supplemental Results

1.1 Rapamycin induces less immunogenic DCs

RNA-seq analysis using DC cells treated with Rapamycin versus vehicle showed that Rapa-M-DC had a total of 139 DEGs (False discovery rate (FDR)<0.05 (the estimated probability that the normalized enrichment score represents a false-positive finding); Fold change (FC) ≥2), 84 genes were down-regulated, and 55 genes were up-regulated when compared to Veh-DC (Supplemental Table 1). We first identified all statistically enriched terms (GO/KEGG terms, canonical pathways; hallmark gene sets); accumulative hypergeometric *p*-values and enrichment factors were calculated and used for filtering. The remaining significant terms were then hierarchically clustered into a tree based on Kappa-statistical similarities among their gene memberships. Then 0.3 kappa score was applied as the threshold to cast the tree into term clusters. Enrichment analysis (Supplemental Fig. 1A) showed the main pathways associated with Rapa-M-DC associated with cytokine-mediated signaling, regulation of cytokine production, TNF signaling via NF-κB (including sphk1, birc3, pde4b, dennd5a, traf1, ifit2, cxcl3, ccl5, ccrl2, il12b, il6, inhba, ptgs2, il1b, and cxcl10) , and IFN-γ production or IFN-γ response genes ((including b2m, parp14, ly6e, mthfd2, cd86, pde4b, mvp, stat2, sppl2a, irf8, ifit2, oas3, xaf1, ifit1, rsad2, ccl5, isg15, il6, oas2, ifi44, casp4, ptgs2, cxcl10 genes), all critical in both innate and adaptive immune response. Additionally, pathway analysis using Reactome (www.reactome.org) showed IL10 signaling as a major changing pathway (FDR of $5.87X10^{-04}$). Then a subset of representative terms from this cluster was selected and converted to a network layout. More specifically, each term is represented by a circle node, where its size is proportional to the number of input genes that fall into that term, and its color represents its cluster identity (i.e., nodes of the same color belong to the same cluster). Terms with a similarity score > 0.3 were linked by an edge (the thickness of the edge represents the similarity score). The network was visualized with Cytoscape (v3.1.2) using a “force-directed” layout and with edge bundled for clarity. One term from each

cluster was selected to have its term description shown as label. As shown in the Supplemental Fig. 1B, the DEGs that were associated within pathways using gene enrichment approaches were significantly linked in networks, with most of them showing a direct effect in immune response.

2.Supplemental Materials and Methods

2.1 RNA-seq and Analysis

400 ng of total RNA was used to generate libraries for RNA sequencing using NEB Next Ultra II-Dimensional RNA-seq library prep kit for Illumina (E7765). Briefly, poly A enrichment was performed using NEBNext Poly(A) enrichment module (E7490). First strand and second strand synthesis were done as recommended in the manual. After end repair and adaptor ligation, libraries were indexed, and PCR amplified. Final libraries were quantified by qubit and Agilent bioanalyzer, combined, and sequenced at Oklahoma Medical Research Foundation Clinical Genomics Center using NextSeq500. RNA-Seq Analysis: Libraries were sequenced with 72 bp paired end reads for 2 conditions with 3 biological replicates each. Sequencing reads were first processed (trimming of 5 nt from 5' end) and quality filtered (removal of reads with phred score < 28 in 80% of the read length) using fastx_toolkit. Trimmed and filtered reads were aligned to mm38 reference genome using bowtie2 with mapping statistics ranging from 88.9%-93.6% for 6 samples[1]. Counts per gene were obtained with Ht seq-count[2]. DESeq2 was used to identify differentially expressed genes for comparisons between Veh/Veh and Rapa/Veh[3].

2.2 BMDC staining and FACS

WT DCs were isolated and propagated for 8 days in presence of GMCSF, total of 3 treatments. Single or multiple Rapamycin (10 ng/ml) treated DCs were used for flow cytometry and imaging using mitochondrial membrane potential probe, JC-1 (ThermoFisher, Waltham, MA). Eight-day old Veh-DCs, Rapa(M)-DCs and Rapa(S)-DCs were grown on cover slips and allowed to attach over-night before staining with Hoechst (5 µg/ml) and JC-1 (5 µM) for 30 mins at 37°C. Some

cells were treated with Hoechst and JC-1 at same concentrations for flow cytometry analysis. Fluorescence images were taken using Nikon Eclipse Ti2 microscope.

Supplemental Figure Legend

Supplemental Figure 1. Bioinformatics analysis suggests propagation of BMDCs in presence of Rapamycin induces anti-inflammatory phenotype. **(A)** Enrichment Analysis indicating main pathways associated with treatment of DCs with Rapamycin-M that include cytokine-mediated signaling, regulation of cytokine production, TNF signaling, and IFN gamma production. **(B)** Network analysis using Cytoscape (v3.1.2) with “force-directed” layout and with edge bundled for clarity. One term from each cluster is selected to have its term description shown as label. The DEGs that were associated with pathways using gene enrichment approaches (as shown in 1A) were significantly linked in networks.

Supplemental Figure 2. Bone marrow cells were stained with JC-1 for flow cytometry and immunofluorescence imaging. **(A)** Veh-DC, Rapa-M-DC, and Rapa-S-DC were labeled with JC-1 (5 μM) and Hoechst (5 μg/ml) for immunofluorescence imaging. Scale bar = 100 μm. **(B)** Flow cytometry analysis of the Veh-DC, Rapa-M-DC, and Rapa-S-DC were labeled with JC-1 (5 μM) and Hoechst (5 μg/ml).

Supplemental Figure 3. TUNEL analysis of mice treated with either NC (no cells) or Veh-DC, Rapa-M-DC or Rapa-S-DC or Mito-DC in sham and kidney IRI. Representative images are shown. Scale bar = 200 μm.

Supplemental Figure 4. Original western blot images of OxPhos cocktail for blots in Figure 6I (top, Veh/Veh, Veh/LPS (6 hrs), and Veh/LPS (24 hrs).

Supplemental Figure 5. Original western blot images of GAPDH for blots in Figure 6I (top, Veh/Veh, Veh/LPS (6 hrs), and Veh/LPS (24 hrs). The GAPDH blots were cut prior hybridization with antibodies.

Supplemental Figure 6. Original western blot images of OxPhos cocktail for blots in Figure 6I (top, Mito/Veh, Mito/LPS (6 hrs), and Mito/LPS (24 hrs).

Supplemental Figure 7. Original western blot images of GAPDH for blots in Figure 6I (top, Mito/Veh, Mito/LPS (6 hrs), and Mito/LPS (24 hrs). The GAPDH blots were cut prior hybridization with antibodies.

Supplemental References

1. Langmead, B.; Salzberg, S.L. Fast gapped-read alignment with Bowtie 2. *Nat Methods* **2012**, *9*, 357-359, doi:10.1038/nmeth.1923.
2. Anders, S.; Pyl, P.T.; Huber, W. HTSeq--a Python framework to work with high-throughput sequencing data. *Bioinformatics* **2015**, *31*, 166-169, doi:10.1093/bioinformatics/btu638.
3. Love, M.I.; Huber, W.; Anders, S. Moderated estimation of fold change and dispersion for RNA-seq data with DESeq2. *Genome Biol* **2014**, *15*, 550, doi:10.1186/s13059-014-0550-8.

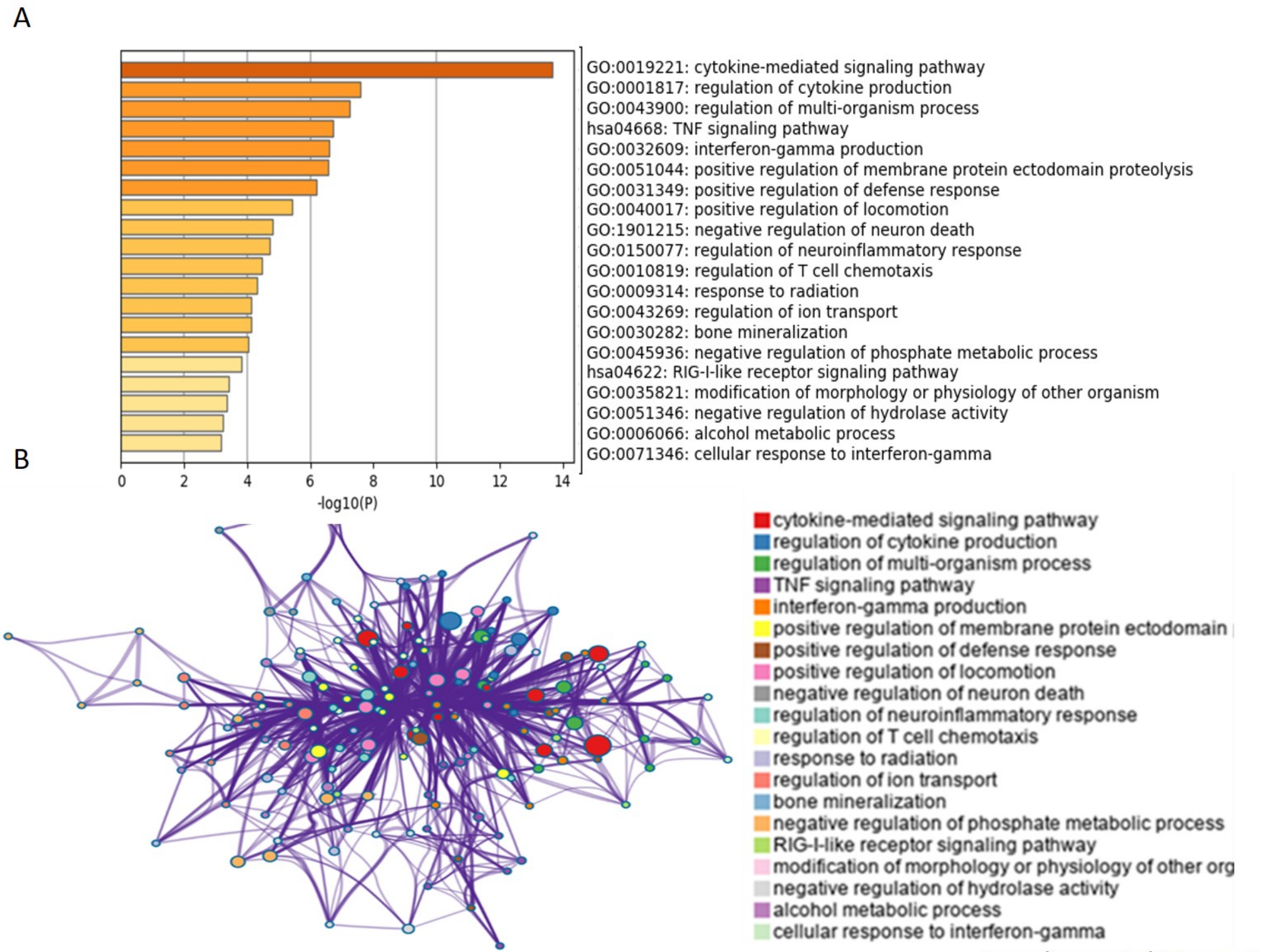

Supplemental Figure 1

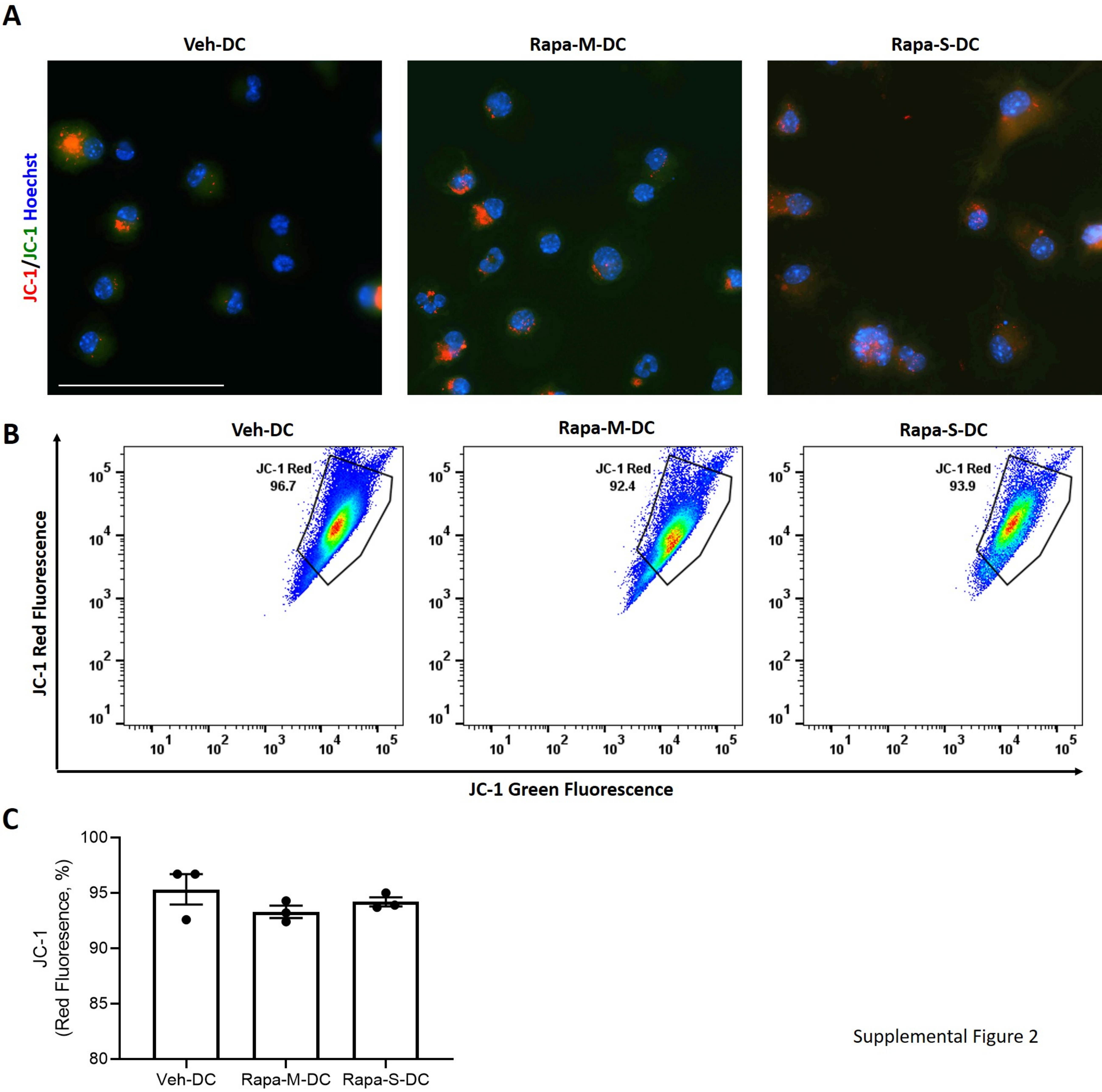

Supplemental Figure 2

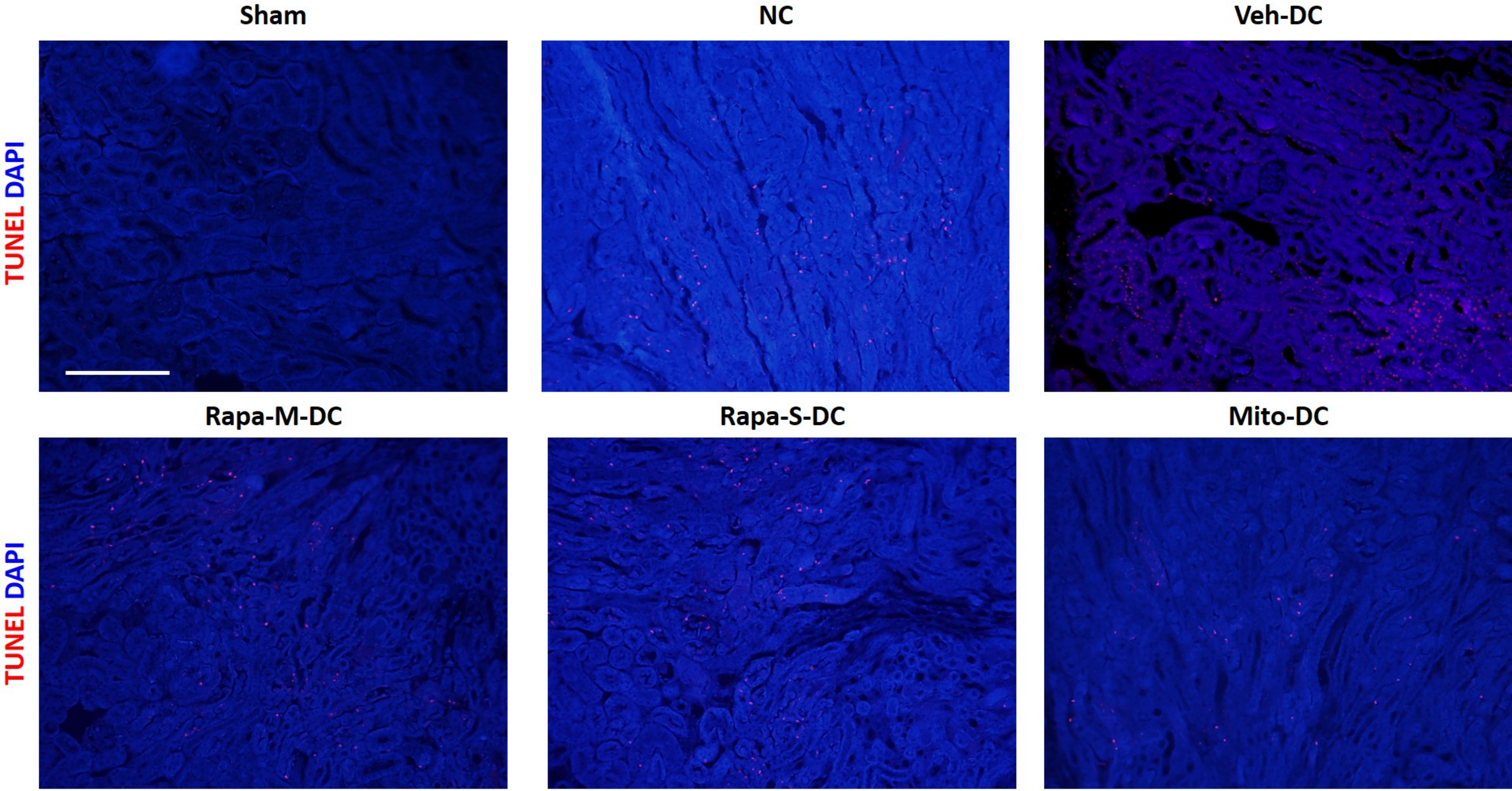

Supplemental Figure 3

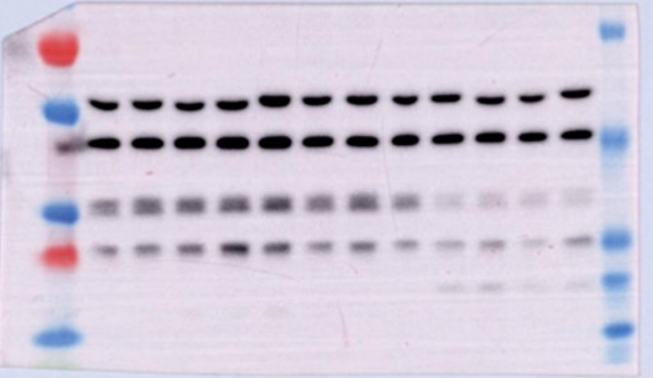

Supplemental Figure 4

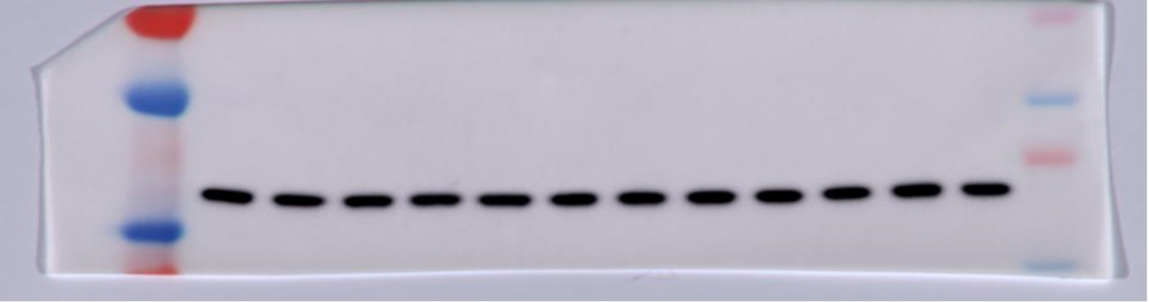

Supplemental Figure 5

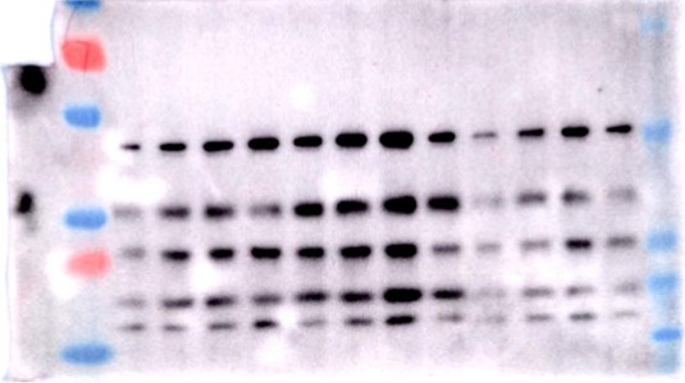

Supplemental Figure 6

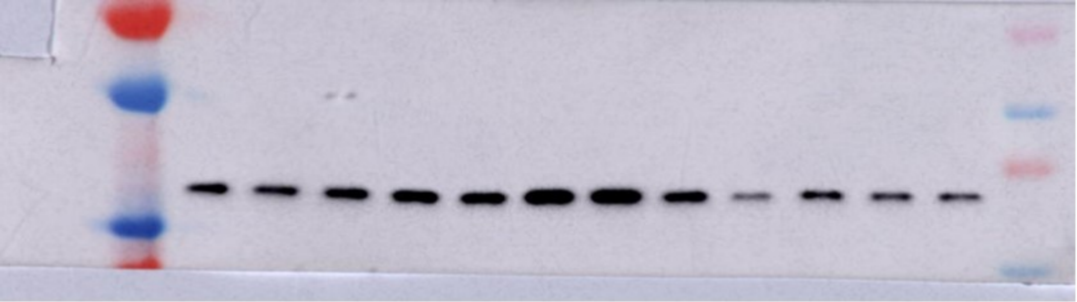

Supplemental Figure 7

Supplemental Table 1: Significantly regulated differentially expressed genes in multiple Rapamycin treated DCs (Rapa-M-DC) compared to vehicle treated DCs.

Ensemble Id	GeneNames	baseMean	log2 FoldChange	lfcSE	stat	p-value	p-adj
ENSMUSG00000030590	Fam98c	234.62	2.96	0.44	6.66	0.00000	0.00000
ENSMUSG00000047369	Dnah14	214.62	2.38	0.41	5.76	0.00000	0.00003
ENSMUSG00000087135	Gm16096	191.30	-3.36	0.64	-5.29	0.00000	0.00025
ENSMUSG00000023988	Bysl	914.88	1.92	0.38	5.01	0.00000	0.00059
ENSMUSG00000031072	LTO1	406.23	2.79	0.56	5.00	0.00000	0.00059
ENSMUSG00000033717	Adra2a	51.36	3.65	0.73	5.02	0.00000	0.00059
ENSMUSG00000054006	D630008O14Rik	42.44	2.66	0.54	4.94	0.00000	0.00064
ENSMUSG00000086742	Gm16201	154.18	1.65	0.34	4.93	0.00000	0.00064
ENSMUSG00000090009	Gm16282	63.51	2.26	0.47	4.85	0.00000	0.00085
ENSMUSG00000046806	Cyren	687.27	-2.76	0.58	-4.77	0.00000	0.00115
ENSMUSG00000114375	Gm31544	57.26	-3.18	0.68	-4.67	0.00000	0.00169
ENSMUSG00000103857	Gm37249	43.67	2.03	0.44	4.58	0.00000	0.00236
ENSMUSG00000090266	Mettl23	1,303.40	1.23	0.27	4.56	0.00001	0.00240
ENSMUSG00000041515	Irf8	29.12	-3.38	0.74	-4.54	0.00001	0.00252
ENSMUSG00000028249	Sdcbp	96.79	1.50	0.33	4.48	0.00001	0.00293
ENSMUSG00000087633	Gm14455	64.49	3.58	0.80	4.48	0.00001	0.00293
ENSMUSG00000114871	Gm21370	63.84	-3.13	0.71	-4.43	0.00001	0.00339
ENSMUSG00000085457	1110046J04Rik	12.38	-3.85	0.88	-4.40	0.00001	0.00375
ENSMUSG00000004296	Il12b	51.71	-5.65	1.31	-4.32	0.00002	0.00481
ENSMUSG00000004707	Ly9	98.43	2.56	0.60	4.30	0.00002	0.00481
ENSMUSG00000029816	Gpnmb	28.56	-3.29	0.77	-4.30	0.00002	0.00481
ENSMUSG00000047293	Gpr15	68.55	3.52	0.82	4.31	0.00002	0.00481
ENSMUSG00000041324	Inhba	58.58	-5.87	1.37	-4.28	0.00002	0.00500
ENSMUSG00000047721	Bola2	453.77	1.82	0.44	4.15	0.00003	0.00834
ENSMUSG00000040963	Asgr2	16.60	2.55	0.62	4.13	0.00004	0.00881
ENSMUSG00000035042	Ccl5	113.00	-4.60	1.13	-4.06	0.00005	0.01054
ENSMUSG00000103175	Gm37169	14.66	4.85	1.19	4.07	0.00005	0.01054
ENSMUSG00000108219	Gm44101	9.86	2.24	0.55	4.07	0.00005	0.01054
ENSMUSG00000038415	Foxq1	13.23	-3.84	0.95	-4.05	0.00005	0.01061
ENSMUSG00000069516	Lyz2	128.78	-2.73	0.67	-4.05	0.00005	0.01061
ENSMUSG00000046338	Gpat2	9.81	2.74	0.68	4.04	0.00005	0.01070
ENSMUSG00000101144	Gm29054	18.89	-3.64	0.91	-4.00	0.00006	0.01229
ENSMUSG00000026358	Rgs1	40.37	-4.98	1.25	-3.98	0.00007	0.01262
ENSMUSG00000040026	Saa3	33.90	-6.72	1.69	-3.97	0.00007	0.01311
ENSMUSG00000059659	Gm10069	50.40	1.63	0.41	3.96	0.00008	0.01340
ENSMUSG00000098645	Gm27201	9.86	2.82	0.72	3.94	0.00008	0.01413
ENSMUSG00000037242	Clic4	26.96	-2.18	0.56	-3.92	0.00009	0.01461
ENSMUSG00000029561	Oasl2	23.56	-4.18	1.08	-3.89	0.00010	0.01636
ENSMUSG00000097272	Gm26649	394.09	1.50	0.39	3.88	0.00010	0.01649

ENSMUSG00000002985	Apoe	10.99	-4.54	1.19	-3.82	0.00013	0.01694
ENSMUSG00000019822	Smpd2	474.25	1.48	0.39	3.83	0.00013	0.01694
ENSMUSG00000021273	Fdft1	210.81	-2.56	0.67	-3.83	0.00013	0.01694
ENSMUSG00000021578	Ccdc127	10.94	-4.96	1.29	-3.85	0.00012	0.01694
ENSMUSG00000025407	Gli1	71.90	1.61	0.42	3.84	0.00012	0.01694
ENSMUSG00000029815	Malsu1	11.82	-4.32	1.12	-3.85	0.00012	0.01694
ENSMUSG00000031390	Avpr2	247.07	1.70	0.44	3.83	0.00013	0.01694
ENSMUSG00000038583	Pln	31.08	2.06	0.54	3.82	0.00013	0.01694
ENSMUSG00000044867	Gimap1os	24.23	3.22	0.84	3.82	0.00014	0.01694
ENSMUSG00000052861	Dnah6	27.99	-4.73	1.24	-3.83	0.00013	0.01694
ENSMUSG00000106933	Gm43621	90.46	1.73	0.45	3.81	0.00014	0.01721
ENSMUSG00000001175	Calm1	45.23	-2.50	0.66	-3.78	0.00016	0.01818
ENSMUSG00000074354	Arhgap20os	31.26	2.50	0.66	3.78	0.00015	0.01818
ENSMUSG00000085088	4931413K12Rik	49.58	1.75	0.46	3.79	0.00015	0.01818
ENSMUSG00000052477	C130026I21Rik	12.43	-3.13	0.83	-3.76	0.00017	0.01902
ENSMUSG00000100763	Gm28777	8.00	2.56	0.68	3.73	0.00019	0.02110
ENSMUSG00000020053	Igf1	10.70	-6.12	1.65	-3.71	0.00021	0.02172
ENSMUSG00000020641	Rsad2	49.57	-4.18	1.13	-3.71	0.00021	0.02172
ENSMUSG00000039697	Ncoa7	19.63	-3.37	0.91	-3.71	0.00020	0.02172
ENSMUSG00000073489	Ifi204	16.58	-3.20	0.86	-3.71	0.00021	0.02172
ENSMUSG00000026969	Fam166a	132.90	1.64	0.44	3.70	0.00022	0.02226
ENSMUSG00000032487	Ptgs2	25.57	-6.69	1.82	-3.68	0.00023	0.02307
ENSMUSG00000023908	Pkmyt1	32.50	1.71	0.46	3.68	0.00023	0.02327
ENSMUSG00000058470	Gm8369	8.02	-5.72	1.57	-3.64	0.00028	0.02557
ENSMUSG00000084137	Gm9085	10.79	3.01	0.83	3.64	0.00028	0.02557
ENSMUSG00000106082	4930404A12Rik	268.96	1.54	0.42	3.63	0.00028	0.02557
ENSMUSG00000106478	Gm36551	50.89	2.07	0.57	3.65	0.00026	0.02557
ENSMUSG00000111429	Gm32511	26.77	3.28	0.90	3.64	0.00027	0.02557
ENSMUSG00000017493	Igfbp4	10.75	-5.39	1.49	-3.63	0.00029	0.02559
ENSMUSG00000109251	E230032D23Rik	41.87	-2.43	0.67	-3.63	0.00029	0.02559
ENSMUSG00000027398	Il1b	37.30	-6.75	1.87	-3.61	0.00031	0.02634
ENSMUSG00000032661	Oas3	15.22	-3.76	1.04	-3.61	0.00031	0.02634
ENSMUSG00000040483	Xaf1	18.21	-3.93	1.09	-3.61	0.00031	0.02634
ENSMUSG00000062515	Fabp4	7.08	-4.42	1.23	-3.59	0.00032	0.02731
ENSMUSG00000001056	Nhp2	47.60	1.97	0.55	3.57	0.00035	0.02878
ENSMUSG00000018796	Acsl1	15.47	-4.12	1.16	-3.56	0.00037	0.02878
ENSMUSG00000040738	Ints8	34.18	2.18	0.61	3.57	0.00036	0.02878
ENSMUSG00000099569	Gm18301	18.04	-6.41	1.80	-3.57	0.00036	0.02878
ENSMUSG00000100280	Gm28417	203.48	1.07	0.30	3.57	0.00036	0.02878
ENSMUSG00000031537	Ikbkb	25.51	-3.47	0.97	-3.56	0.00037	0.02894
ENSMUSG00000023913	Pla2g7	14.57	-2.53	0.72	-3.54	0.00040	0.03064
ENSMUSG00000030402	Ppm1n	19.37	-6.18	1.75	-3.54	0.00041	0.03079
ENSMUSG00000003746	Man1a	12.61	-4.39	1.25	-3.51	0.00045	0.03187

ENSMUSG00000020397	Med7	72.72	-4.72	1.34	-3.52	0.00043	0.03187
ENSMUSG00000022901	Cd86	37.20	-2.84	0.81	-3.52	0.00044	0.03187
ENSMUSG00000069184	Zfp72	8.49	-4.31	1.23	-3.51	0.00045	0.03187
ENSMUSG00000074269	Rec114	72.84	1.23	0.35	3.51	0.00045	0.03187
ENSMUSG00000118361	AC132320.1	15.35	-4.48	1.28	-3.51	0.00045	0.03187
ENSMUSG00000084864	1700027A07Rik	148.99	1.62	0.46	3.50	0.00046	0.03205
ENSMUSG00000085315	A430018G15Rik	13.13	2.02	0.58	3.50	0.00046	0.03205
ENSMUSG00000031393	Mecp2	11.53	-3.79	1.08	-3.49	0.00047	0.03238
ENSMUSG00000022747	St3gal6	169.14	-1.89	0.54	-3.49	0.00048	0.03260
ENSMUSG00000085192	Gm12195	27.88	1.74	0.50	3.49	0.00049	0.03260
ENSMUSG00000087698	Gm13031	15.33	2.07	0.60	3.47	0.00051	0.03377
ENSMUSG00000028037	Ifi44	8.07	-6.26	1.81	-3.47	0.00052	0.03410
ENSMUSG00000040033	Stat2	32.98	-3.25	0.94	-3.45	0.00056	0.03589
ENSMUSG00000026875	Traf1	31.45	-3.57	1.04	-3.44	0.00058	0.03725
ENSMUSG00000026201	Stk16	48.66	1.83	0.53	3.44	0.00059	0.03731
ENSMUSG00000020527	Myo19	34.08	1.25	0.36	3.43	0.00061	0.03746
ENSMUSG00000045962	Wnk1	29.25	-2.82	0.82	-3.42	0.00062	0.03746
ENSMUSG00000060509	Xcr1	25.96	-1.79	0.52	-3.43	0.00061	0.03746
ENSMUSG00000086430	4930551O13Rik	25.76	-3.33	0.97	-3.42	0.00062	0.03746
ENSMUSG00000107560	Gm44199	41.41	1.39	0.41	3.42	0.00062	0.03760
ENSMUSG00000027611	Procr	49.66	-4.95	1.45	-3.42	0.00063	0.03773
ENSMUSG00000039109	F13a1	7.46	-5.75	1.68	-3.41	0.00064	0.03775
ENSMUSG00000026827	Gpd2	14.54	-3.65	1.07	-3.40	0.00068	0.03813
ENSMUSG00000034855	Cxcl10	10.10	-6.88	2.02	-3.41	0.00066	0.03813
ENSMUSG00000040270	Bach2	22.66	-5.05	1.48	-3.41	0.00066	0.03813
ENSMUSG00000042508	Dmtf1	165.98	1.39	0.41	3.40	0.00068	0.03813
ENSMUSG00000090200	1700025N21Rik	98.87	2.15	0.63	3.40	0.00068	0.03813
ENSMUSG00000033538	Casp4	8.86	-6.38	1.88	-3.39	0.00070	0.03881
ENSMUSG00000035168	Tanc1	8.92	-5.32	1.57	-3.39	0.00071	0.03881
ENSMUSG00000086507	Adap2os	42.78	-1.97	0.58	-3.38	0.00071	0.03881
ENSMUSG00000107143	Gm6598	28.54	-1.16	0.34	-3.39	0.00070	0.03881
ENSMUSG00000022781	Pak2	15.02	-3.32	0.98	-3.38	0.00073	0.03941
ENSMUSG00000087067	Gm11532	91.55	-3.04	0.90	-3.37	0.00076	0.04081
ENSMUSG00000022023	Wbp4	19.43	-2.30	0.68	-3.35	0.00079	0.04096
ENSMUSG00000024805	Pcgf5	22.01	-2.96	0.88	-3.36	0.00078	0.04096
ENSMUSG00000085684	4930469K13Rik	19.72	1.37	0.41	3.36	0.00079	0.04096
ENSMUSG00000090863	A530084C06Rik	10.30	-2.45	0.73	-3.36	0.00079	0.04096
ENSMUSG00000079293	Clec7a	19.07	-2.40	0.72	-3.35	0.00080	0.04115
ENSMUSG00000067851	Arfgef1	11.44	-3.04	0.91	-3.35	0.00081	0.04123
ENSMUSG00000079036	Alkbh1	60.89	-1.99	0.60	-3.34	0.00083	0.04155
ENSMUSG00000005370	Msh6	17.92	1.63	0.49	3.32	0.00091	0.04533
ENSMUSG00000037997	Parp11	10.75	-5.00	1.51	-3.31	0.00093	0.04618
ENSMUSG00000028599	Tnfrsf1b	28.97	-2.81	0.85	-3.31	0.00095	0.04661

ENSMUSG00000087444	Gm5475	13.21	-5.86	1.78	-3.30	0.00097	0.04709
ENSMUSG00000073725	Lmbrd1	9.19	-6.58	2.00	-3.29	0.00099	0.04774
ENSMUSG00000030144	Clec4d	9.77	-3.02	0.92	-3.29	0.00101	0.04829
ENSMUSG00000037613	Tnfrsf23	11.64	-2.42	0.74	-3.29	0.00101	0.04829
ENSMUSG00000002844	Adprh	41.91	-4.94	1.51	-3.27	0.00107	0.04871
ENSMUSG00000022587	Ly6e	31.95	-2.74	0.84	-3.26	0.00110	0.04871
ENSMUSG00000022876	Samsn1	11.54	-2.05	0.63	-3.28	0.00105	0.04871
ENSMUSG00000026525	Opn3	16.65	3.57	1.09	3.28	0.00103	0.04871
ENSMUSG00000034377	Tulp4	756.93	1.18	0.36	3.28	0.00104	0.04871
ENSMUSG00000035692	Isg15	17.33	-4.85	1.49	-3.27	0.00108	0.04871
ENSMUSG00000040829	Zmynd15	1,742.13	-2.10	0.64	-3.27	0.00108	0.04871
ENSMUSG00000049362	Olfr173	35.77	1.86	0.57	3.27	0.00109	0.04871
ENSMUSG00000060802	B2m	98.81	-1.95	0.60	-3.27	0.00109	0.04871
ENSMUSG00000096980	Gm26526	74.32	-1.21	0.37	-3.26	0.00113	0.04982

Supplemental Table 2: Relative changes in genes related to Autophagy, Cytokines, Inflammation in Vehicle and Rapamycin (Rapa-S or Rapa-M) DCs with and without LPS after 24 hrs. The genes after LPS indicated in green were down-regulated and in red were up-regulated compared to respective vehicle treatment.

Gene	Veh/Veh	Veh/LPS	Rapa-M/Veh	Rapa-M/LPS	Rapa-S/Veh	Rapa-S/LPS
Autophagy						
Beclin	$2.5x10^{-2} \pm 1.0x10^{-3}$	$7.6\ x10^{-3} \pm 1.7x10^{-3}$	$1.5x10^{-2} \pm 4.3x10^{-3}$	$1.9x10^{-2} \pm 2.2x10^{-3}$	$2.3x10^{-2} \pm 6.2x10^{-3}$	$4.4x10^{-3} \pm 1.5x10^{-3}$
Atg9	$6.4x10^{-3} \pm 5.0x10^{-4}$	$1.1\ x10^{-2} \pm 1.1x10^{-3}$	$1.1x10^{-2} \pm 1.4x10^{-3}$	$1.0x10^{-2} \pm 6.3x10^{-4}$	$1.6x10^{-2} \pm 2.7x10^{-3}$	$1.1x10^{-2} \pm 9.0x10^{-4}$
Atg7	$7.6x10^{-3} \pm 5.0x10^{-4}$	$1.1\ x10^{-2} \pm 1.1x10^{-3}$	$1.2x10^{-2} \pm 6.0x10^{-4}$	$1.0x10^{-2} \pm 1.6x10^{-3}$	$1.6x10^{-2} \pm 7.5x10^{-4}$	$9.7x10^{-3} \pm 6.2x10^{-4}$
Lc3b	$1.4x10^{-3} \pm 1.0x10^{-4}$	$1.2\ x10^{-3} \pm 1.3x10^{-4}$	$7.9x10^{-4} \pm 9.6x10^{-5}$	$1.1x10^{-3} \pm 1.4x10^{-4}$	$7.8x10^{-4} \pm 2.2x10^{-4}$	$2.2x10^{-4} \pm 5.0x10^{-5}$
Lamp2	$7.1x10^{-1} \pm 2.9x10^{-2}$	$3.5\ x10^{-1} \pm 6.3x10^{-2}$	$3.5x10^{-1} \pm 1.1x10^{-1}$	$7.7x10^{-1} \pm 1.8x10^{-1}$	$1.2x10^{+0} \pm 4.9x10^{-2}$	$7.6x10^{-1} \pm 3.2x10^{-2}$
Cytokines						
Il1b	$6.1x10^{-2} \pm 1.9x10^{-2}$	$3.9\ x10^{0} \pm 5.5x10^{-1}$	$3.3x10^{-2} \pm 2.8x10^{-3}$	$1.9x10^{+0} \pm 3.7x10^{-1}$	$7.4x10^{-2} \pm 2.4x10^{-2}$	$1.1x10^{0} \pm 4.5x10^{-1}$
Il12p40	$8.9x10^{-3} \pm 5.0x10^{-4}$	$5.6\ x10^{-1} \pm 1.2x10^{-1}$	$8.5x10^{-3} \pm 1.8x10^{-3}$	$1.4x10^{-1} \pm 2.3x10^{-2}$	$1.6x10^{-2} \pm 1.6x10^{-3}$	$1.5x10^{-1} \pm 5.6x10^{-2}$
Inflammation						
Tlr4	$4.6x10^{-2} \pm 3.8x10^{-3}$	$1.4\ x10^{-2} \pm 1.7x10^{-3}$	$1.8x10^{-2} \pm 4.7x10^{-3}$	$3.8x10^{-2} \pm 7.0x10^{-3}$	$5.0x10^{-2} \pm 1.3x10^{-2}$	$1.3x10^{-2} \pm 3.4x10^{-3}$
Nos2	$2.7x10^{-3} \pm 1.0x10^{-4}$	$4.1\ x10^{-2} \pm 3.5x10^{-3}$	$8.7x10^{-3} \pm 2.3x10^{-3}$	$1.0x10^{-2} \pm 9.5x10^{-4}$	$8.8x10^{-3} \pm 2.1x10^{-3}$	$2.1x10^{-2} \pm 1.7x10^{-3}$
Hif1a	$1.1x10^{-1} \pm 3.2x10^{-2}$	$1.6\ x10^{-1} \pm 2.9x10^{-2}$	$1.3x10^{-1} \pm 8.3x10^{-3}$	$4.3x10^{-2} \pm 6.2x10^{-3}$	$1.4x10^{-1} \pm 3.5x10^{-2}$	$3.1x10^{-2} \pm 6.7x10^{-4}$
Other						
Ho1	$7.2x10^{-2} \pm 5.0x10^{-3}$	$3.2\ x10^{-1} \pm 6.0x10^{-2}$	$2.8x10^{-2} \pm 2.6x10^{-3}$	$7.3x10^{-2} \pm 1.3x10^{-2}$	$6.1x10^{-2} \pm 1.0x10^{-2}$	$6.4x10^{-2} \pm 1.5x10^{-2}$

Supplemental Table 3: Relative changes in genes related to Autophagy, Cytokines, Inflammation in Vehicle and Mitochondria DCs (Mito-DC) with and without LPS after 24 hrs. The genes after LPS indicated in green were down-regulated and in red were up-regulated compared to respective vehicle treatment.

Gene	Veh/Veh	Veh/LPS	Mito/Veh	Mito/LPS
Autophagy				
Beclin	$7.1x10^{-2} \pm 2.6x10^{-2}$	$4.7x10^{-2} \pm 1.5x10^{-2}$	$1.8x10^{-2} \pm 8.1x10^{-3}$	$8.6x10^{-3} \pm 1.9x10^{-3}$
Atg9	$4.7x10^{-3} \pm 2.1x10^{-3}$	$1.9x10^{-3} \pm 5.5x10^{-4}$	$1.4x10^{-4} \pm 8.6x10^{-5}$	$9.5x10^{-2} \pm 3.1x10^{-5}$
Atg7	$6.5x10^{-2} \pm 2.9x10^{-2}$	$2.3x10^{-2} \pm 6.3x10^{-3}$	$9.0x10^{-3} \pm 3.7x10^{-3}$	$5.2x10^{-3} \pm 1.5x10^{-3}$
Lc3b	$1.1x10^{-2} \pm 5.5x10^{-3}$	$5.6x10^{-3} \pm 1.9x10^{-3}$	$1.0x10^{-2} \pm 7.3x10^{-3}$	$3.4x10^{-3} \pm 1.3x10^{-3}$
Lamp2	$2.0x10^{0} \pm 8.0x10^{-1}$	$1.5x10^{0} \pm 4.9x10^{-1}$	$8.4x10^{-1} \pm 5.4x10^{-1}$	$2.0x10^{-1} \pm 1.8x10^{-2}$
Cytokines				
Il1b	$3.9x10^{-2} \pm 1.4x10^{-2}$	$1.5x10^{0} \pm 3.3x10^{-1}$	$2.3x10^{-1} \pm 3.1x10^{-2}$	$4.5x10^{-1} \pm 1.6x10^{-1}$
Il12p40	$1.4x10^{-1} \pm 4.7x10^{-3}$	$6.5x10^{-1} \pm 6.0x10^{-2}$	$8.4x10^{-1} \pm 8.1x10^{-2}$	$1.0x10^{0} \pm 2.6x10^{-2}$
Inflammation				
Tlr4	$2.2x10^{-1} \pm 9.4x10^{-2}$	$7.0x10^{-2} \pm 2.1x10^{-2}$	$1.9x10^{-2} \pm 1.3x10^{-2}$	$4.5x10^{-3} \pm 1.7x10^{-3}$
Nos2	$4.9x10^{-4} \pm 7.2x10^{-5}$	$3.3x10^{-1} \pm 2.4x10^{-2}$	$9.3x10^{-2} \pm 1.0x10^{-2}$	$9.5x10^{-2} \pm 7.2x10^{-3}$
Hif1a	$2.7x10^{-1} \pm 2.8x10^{-2}$	$1.8x10^{-1} \pm 1.6x10^{-2}$	$3.4x10^{-2} \pm 2.6x10^{-3}$	$2.8x10^{-2} \pm 1.4x10^{-3}$
Other				
Ho1	$2.6x10^{-1} \pm 3.6x10^{-2}$	$1.3x10^{0} \pm 1.0x10^{-1}$	$1.6x10^{0} \pm 1.9x10^{-1}$	$7.3x10^{-1} \pm 7.2x10^{-2}$
